# Case of the month 1-2019: CNS high-grade neuroepithelial tumor with *BCOR* alteration 

**DOI:** 10.5414/NP301162

**Published:** 2018-12-11

**Authors:** Christine Haberler, Lilla Reiniger, Hajnalka Rajnai, Ognian Kalev, Ellen Gelpi, Melanie Tamesberger, Torsten Pietsch

**Affiliations:** 1 Institute of Neurology,; 2 Comprehensive Cancer Center, Medical University of Vienna, Vienna, Austria,; 3 I^st^ Department of Pathology and Experimental Cancer Research, Semmelweis University Budapest, Hungary,; 4 Institute of Pathology, Division of Neuropathology, Neuromed Campus, Kepler University Hospital, Johannes Kepler University,; 5 Department of Pediatrics, Kepler University Hospital, Linz, Austria, and; 6 DGNN Brain Tumor Reference Center, Institute of Neuropathology, University of Bonn Medical Center, Bonn, Germany

**Keywords:** CNS high-grade neuroepithelial tumor, *BCOR* alteration

## Abstract

No abstract available.

Central nervous system high-grade neuroepithelial tumor with *BCOR* alteration (CNS HGNET-*BCOR*) has been identified by methylome analysis in 2016 as a distinct molecular CNS tumor entity, which is characterized by an in-frame internal tandem duplication in exon 15 of the *BCOR* gene [14]. The X-linked gene *BCOR* (*BCL6* corepressor) encodes a component of the variant Polycomb repressive complex 1 (PRC1) and may specifically inhibit gene expression. *BCOR* and its paralogue *BCORL1* have been associated with syndromic microphthalmia [8], and mutations have been identified in retinoblastomas, rhabdomyosarcomas, AML as well as in CNS tumors including medulloblastomas and more recently in *H3K27*-mutated diffuse midline gliomas and anaplastic pleomorphic xanthoastrocytomas [3, 7, 10, 11, 13, 17]. *BCOR* in-frame internal tandem duplications in exon 15 and *BCOR-CCNB3/BCOR-MAML1* gene fusion are regarded as specific molecular and key tumor driving event in a subtype of bone sarcoma, clear cell sarcomas of the kidney (CCSK), primitive myxoid mesenchymal tumor of infancy (PMMT), and in endometrial stromal sarcomas [4, 5, 12, 15]. 

Histopathologically, CNS HGNET-*BCOR* tumors were reported as compact tumors with a combination of spindle to oval cells often exhibiting perivascular pseudorosettes, giving the tumors an ependymoma-like appearance in conventional histopathological examinations. Activation of the WNT signaling pathway (nuclear β-catenin immunoreactivity) was observed in 79% of the cases [14], and in a further case also activation of the SHH pathway was found [9]. A detailed histopathological analysis of three pediatric cerebellar tumors with *BCOR* duplication revealed in all cases similar morphological features including uniform ovoid cells with a fine nuclear chromatin structure and a rich arborizing capillary network [2]. Immunohistochemically, all tumors showed a strong NCAM and vimentin immunoreactivity as well as a weaker EGFR expression, whereas all other markers (GFAP, Olig2, desmin, myogenin, synaptophysin, EMA, CD34, NeuN, pan- cytokeratin (AE1/AE3), cytokeratin 8/18, IDH1-R132H, S100 protein, Lin28A, nuclear β-catenin) were not expressed. An anti-BCOR antibody revealed strong nuclear immunolabeling of the cells, which has been also reported in PMMT, round cell sarcomas, and CCSK [1, 12]. Yoshida et al. [16] described 5 cerebellar and 1 temporal tumors emphasizing the uniform character of the tumor cells with a stellate-shape appearance and fibrillary processes. All tumors were strongly vimentin immunoreactive, and a varying expression of Olig2 was reported, whereas GFAP and S100 were only focally encountered. Interestingly, 4 of the 6 tumors displayed also NFP, whereas synaptophysin was present only focally. BCOR protein was expressed in all tumors. Patchy expression of Olig2 and absence of synaptophysin and NFP expression was reported in a further cerebellar CNS HGNET-*BCOR* case [6]. 

We present a case of a CNS HGNET-*BCOR* in a 5-year-old male patient with a large 7 × 6 × 8-cm-sized frontally located, well demarcated tumor (Figure 1A, B). Histopathologically the tumor tissue was compact and highly cellular with rather uniform cells displaying round to ovoid nuclei with fine chromatin structure and a scant, slightly eosinophilic cytoplasm (Figure 1C). Occasionally, a perivascular tumor cell arrangement was detectable. Mitoses were frequently encountered (up to 10 per 1 HPF), the Ki67 labeling index was 40%, and transition into necrosis was present. Glomeruloid vascular proliferations were absent. No reticulin fibers or PAS positivity was found in the Gomori and PAS staining. Immunohistochemically, a strong and widespread vimentin (Figure 1D), EGFR (Figure 1E, F), and NCAM expression was detectable. The majority of tumor cells showed a strong nuclear BCOR immunoreactivity (C-10, 1 : 200, Santa Cruz Biotechnology, Dallas, TX, USA) (Figure 1G). Olig2 was expressed in a fraction of cells (Figure 1H) and a weak cytoplasmic CD99 immunoreactivity was found. GFAP, S100 protein, neurofilament (SMI31 and SMI32), synaptophysin, NeuN, EMA, L1CAM, p65- RelA, Lin28A, and Otx2 stainings were negative. Anti-p53 staining revealed 10% positive nuclei. ATRX, INI1/SMARCB1, and trimethylated H3K27 protein was retained in the nuclei. No mutant H3-K27M or BRAFV600E protein was detectable. Anti-β-catenin staining revealed a strong cytoplasmic staining impeding the evaluation of the nuclear expression. Yet, a faint nuclear immunoreactivity was present and a strong nuclear Yap1 expression similar to that in WNT-activated medulloblastomas was detectable, thus providing some evidence for WNT activation as previously described [14]. DNA was extracted from the FFPE tumor material and PCR using flanking primers, and subsequent Sanger sequencing revealed a large duplication within exon 15 of the *BCOR* gene. The tumor was resected, and control MRI showed a 9-mm contrast enhancing rim at the border of the resection cavity. As the initial diagnosis was glioblastoma, the patient was treated with local radiotherapy with a total dose of 57.6 Gy and received concomitant temozolamide according to the HIT-HGG-2007 protocol. 12 months after diagnosis, a local tumor growth as well as a cerebellar and spinal metastasis occurred. 

To date it remains to be clarified whether tumors with *BCOR* tandem duplication in different locations represent a spectrum of the same tumor entity, similarly as in rhabdoid tumors, and whether CNS HGNET-*BCOR* should be designated CNS *BCOR* sarcoma/mesenchymal tumors as their counterparts in non-CNS locations. The expression of Olig2 indicating neuroepithelial differentiation argues rather against a classical sarcoma phenotype. Tumors with *BCOR* tandem duplications including CNS tumors have been found to be associated with a poor prognosis [2, 5, 14]. A correct diagnosis is important to expand the knowledge on molecular, pathological, and clinical features of these rare malignant tumors, which are not yet included in the current WHO classification of CNS tumors as a distinct entity, and to develop new treatment approaches. Diagnosis of a CNS HGNET-*BCOR* should be considered in malignant tumors reminiscent of ependymoma or glioblastoma without convincing expression of glial markers, and in cerebellar tumors reminiscent of medulloblastoma/embryonal tumors. Strong nuclear BCOR immunoreactivity may be helpful to guide the diagnosis. However, according to the authors personal unpublished experience BCOR immunoreactivity is not specific for HGNET-*BCOR* tumors and may be also encountered in other CNS tumors. Therefore, the diagnosis of a CNS HGNET-*BCOR* tumor should always be confirmed by molecular genetic analyses. 

**Figure 1.**
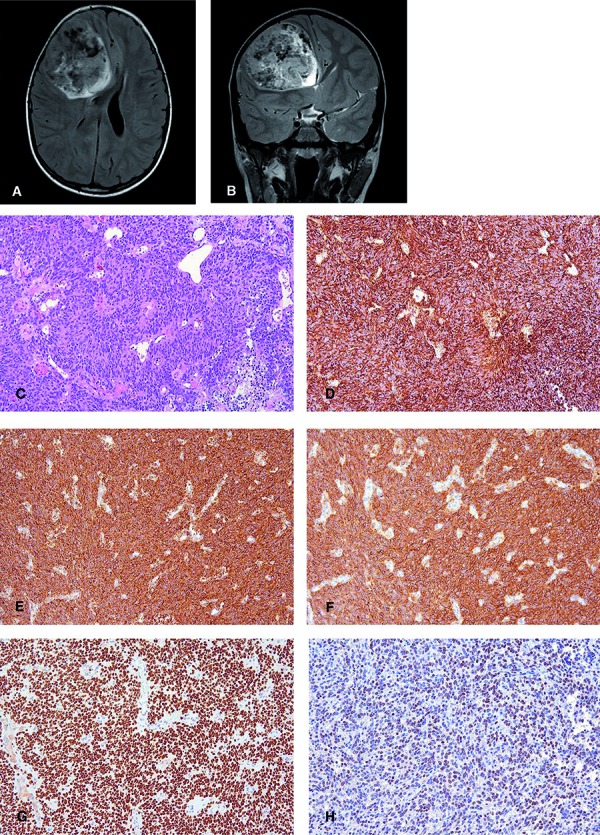
A: Axial FLAIR sequence shows an inhomogeneous space-occupying lesion in the right region displacing the adjacent structures, compressing the right lateral ventricle, and obliterating the foramen of Monroe leading to a consecutive widening of the left lateral ventricle. B: Coronal T2-weighted image emphasizes the hyperintense cystic parts of the lesion. Flow void phenomena, corresponding to abnormal vessels are seen intralesionally on both sequences. C: H & E staining shows a cellular compact tumor with uniform cells displaying round to ovoid nuclei. Widespread intense immunoreactivity for (D) vimentin, (E) EGFR, and (F) Bcl2 was detectable. G: A widespread and strong nuclear expression of BCOR was present. H: Olig2 was expressed in a fraction of cells (Original magnification: C – H, × 200).
